# Supplementary material for: Bridges or Barriers? Cross-boundary communication and governance mismatches in co-managed protected areas
Source: PLoS One. 2026 Apr 6;21(4):e0342438. doi: 10.1371/journal.pone.0342438 (PMC13052881; doi:10.1371/journal.pone.0342438)
Supplement: S1 File — (DOCX) [file pone.0342438.s001.docx]

# Supporting Information

## **A** Further information on the selection of social groups for SNA

Careful selection of who will be included in the network analyses is crucial to portraying representative, non-biased networks (de Juan et al., 2023)^^[[1]](#footnote-2)^^. Some level of bias could not be avoided in this study, e.g., by excluding groups in which members of the board - and potential interviewees - had been allegedly found guilty of criminal charges. However, from the beginning, attention and effort were put into the construction of the list of people to be surveyed considering a careful and unbiased group selection. A List and further description of all groups we included in our study are provided in Table A1 below. 1.

Table A1. Groups and organizations that 1) were or had been part of, 2) had a strong interest in being part of, or 3) would at least like to have their interests considered by the management council of protected areas in the Bragança region, Pará, North Brazil. Members of these groups and organizations were interviewed between November and December 2022. Bold text: Term used in this study for the described groups and organizations.

| Group/ Organization | Area of interest | Group/ Organization type | Local name | Observation | website |
| --- | --- | --- | --- | --- | --- |
| **Administration agency** | regional | (Federal) Government | ICMBio | The RESEXs are managed by an Integrated Management Body (locally known as “NGI”), created in 2018 after the four local administration offices were merged into one | www.gov.br/icmbio/pt-br |
| Non-governmental organization RARE | regional | **NGO** | RARE | This NGO has been working in the region since 2017 and has focused its work on fisheries diagnostics and capacity building to strengthen the management councils of the RESEXs on the Brazilian coast.^^[[2]](#footnote-3)^^ | www.rare.org |
| **Federal Monitoring Agency** | regional | (Federal) Government | Ibama | The “Instituto Brasileiro do Meio Ambiente e dos Recursos Naturais Renováveis” is a federal agency connected to Brazil’s Ministry of Environment. | www.gov.br/ibama/pt-br |
| State-level Department of Environment and the Department for Agricultural and Fisheries Development (**Province Department)** | regional | (State) Government | SEMAS and SEDAP | Department for Environment and Sustainability (SEMAS) and Department for Agricultural and Fisheries Development (SEDAP) | [www.semas.pa.gov.br](http://www.semas.pa.gov.br/)  <https://www.sedap.pa.gov.br/> |
| National-level confederation of marine extractive reserves (**RESEX Confederation**) | regional | Civil Society | CONFREM | The “Comissão Nacional de Fortalecimento das Reservas Extrativistas e Povos Tradicionais Extrativistas Costeiros e Marinhos” is a group that brings together the AUREMs from all the country’s marine RESEXs. | www.confrem.wordpress.com |
| **Academia** | regional | Academia | Universidades (UFPA, UEPA), Institutos de Pesquisa (IFPA, Emílio Goeldi) | Universities and research institutes are usually funded by the federal government but have an independent role in the region. | UFPA (<https://ufpa.br/>)  , UEPA (<https://www.uepa.br/> ), IFPA (<https://ifpa.edu.br/>), , Emílio Goeldi ([www.museu-goeldi.br](http://www.museu-goeldi.br/)  ) |
| Navy | regional | (Federal) Government |  | Not part of the civil government but rather a body of the State apparatus. | https://www.marinha.mil.br/ |
| Town-level Department of Environment and the town-level Department of Fisheries (or agriculture) – **Town Department** | local | (Municipal) Government | SEMMA | City halls have the equivalent of the state-level Department of Environment (SEMA) and sometimes also a Fisheries Department. | Tr:  <https://tracuateua.pa.gov.br/portal-da-transparencia/estrutura-organizacional/secretaria-municipal-de-meio-ambiente/>  and  <https://tracuateua.pa.gov.br/portal-da-transparencia/estrutura-organizacional/secretaria-municipal-de-agricultura/>  Br: <https://braganca.pa.gov.br/secretarias-e-orgaos/secretaria-municipal-de-meio-ambiente/> and <https://braganca.pa.gov.br/secretaria-municipal-de-aquicultura-e-pesca/>  AC:  <https://augustocorrea.pa.gov.br/portal-da-transparencia/estrutura-organizacional/secretaria-municipal-de-meio-ambiente/>  and  https://augustocorrea.pa.gov.br/portal-da-transparencia/estrutura-organizacional/secretaria-municipal-de-agricultura/  Vs:  <http://www.viseu.pa.gov.br/secretaria-municipal-de-meio-ambiente/>  and  <http://www.viseu.pa.gov.br/secretaria-municipal-de-pesca/> |
| The users’ associations (**AUREMs**) | local | Civil Society | AUREMs or ASSUREMs | Each RESEX has its AUREM. | https://mapaosc.ipea.gov.br/ |
| **Deliberative Councils** | local | Civil Society | Conselhos deliberativos | Each RESEX has its council. | no webpage available |
| **Agricultural Assistance** public company | mixed | (State) Government | Empresa de Assistência Técnica e Extensão Rural (EMATER) | Indirect administration public company that provides technical assistance, extension, education, and communication services to the agricultural sector. In Pará state, it is linked to SEDAP (see above). It has offices in each region. Therefore, each Agricultural Assistance office has specific interests in each of the RESEXs but the offices are connected and function as a regional team. | https://emater.pa.gov.br/ |

## **B** Interview guideline

Table B1: Interview guide to access functioning and co-management of four neighboring extractive reserves (RESEX) in the Bragança region, Pará, North Brazil.

| Questions | Possible follow-up questions | Topics discussed |
| --- | --- | --- |
| How is the management of the RESEX? | Do you know about the other RESEXs and their management? | role of the Administration Agency  role of the users, especially the AUREMs  tensions, challenges |
| Are there organizations working to build bridges between the RESEXs? | What could be done to improve joint action between RESEXs? | role of the NGO  role and performance of NGI  integrated management (between the four RESEXs) |
| Is there communication and collaboration between RESEXs? | Why? Why not? What could be done to enhance inter-RESEX communication and collaboration? | role of the councils  integrated management (between the four RESEXs) |
| Are there any successful communication or collaboration projects in the RESEX? | Can you give examples?  Why do you consider them to be/have been successful? | role of the NGO  role and performance of NGI  integrated management (between the four RESEXs) |
| Do you know the NGI? | What is its role? |  |

## **C** Gephi layout settings


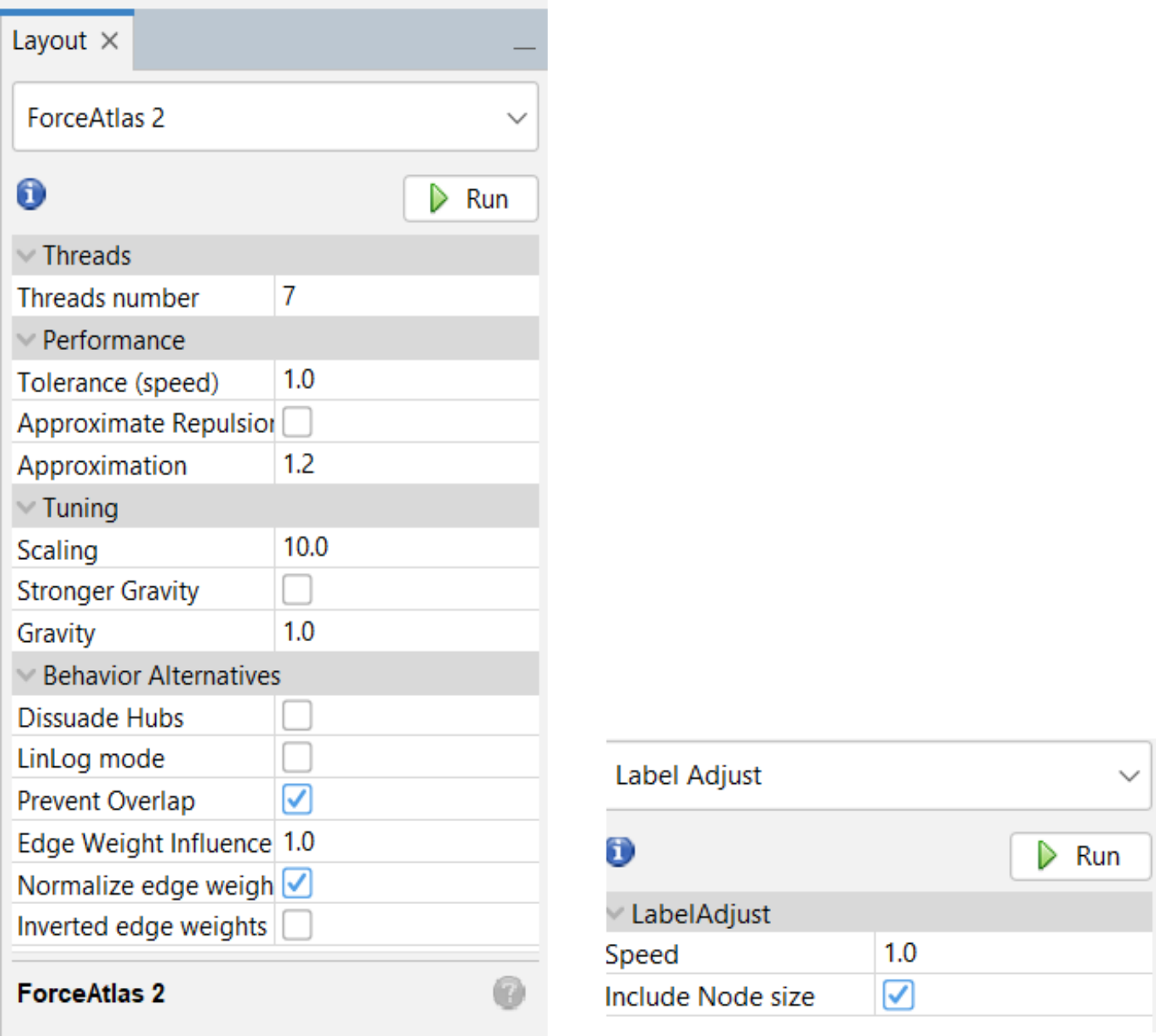


Fig C1: Gephi Layout settings (Bastian M., Heymann S., Jacomy M. (2009). ***Gephi: an open source software for exploring and manipulating networks.*** International AAAI Conference on Weblogs and Social Media.) to visualize the crossboundary governance network of four neighboring administrative regions on the coast of Pará, North Brazil.


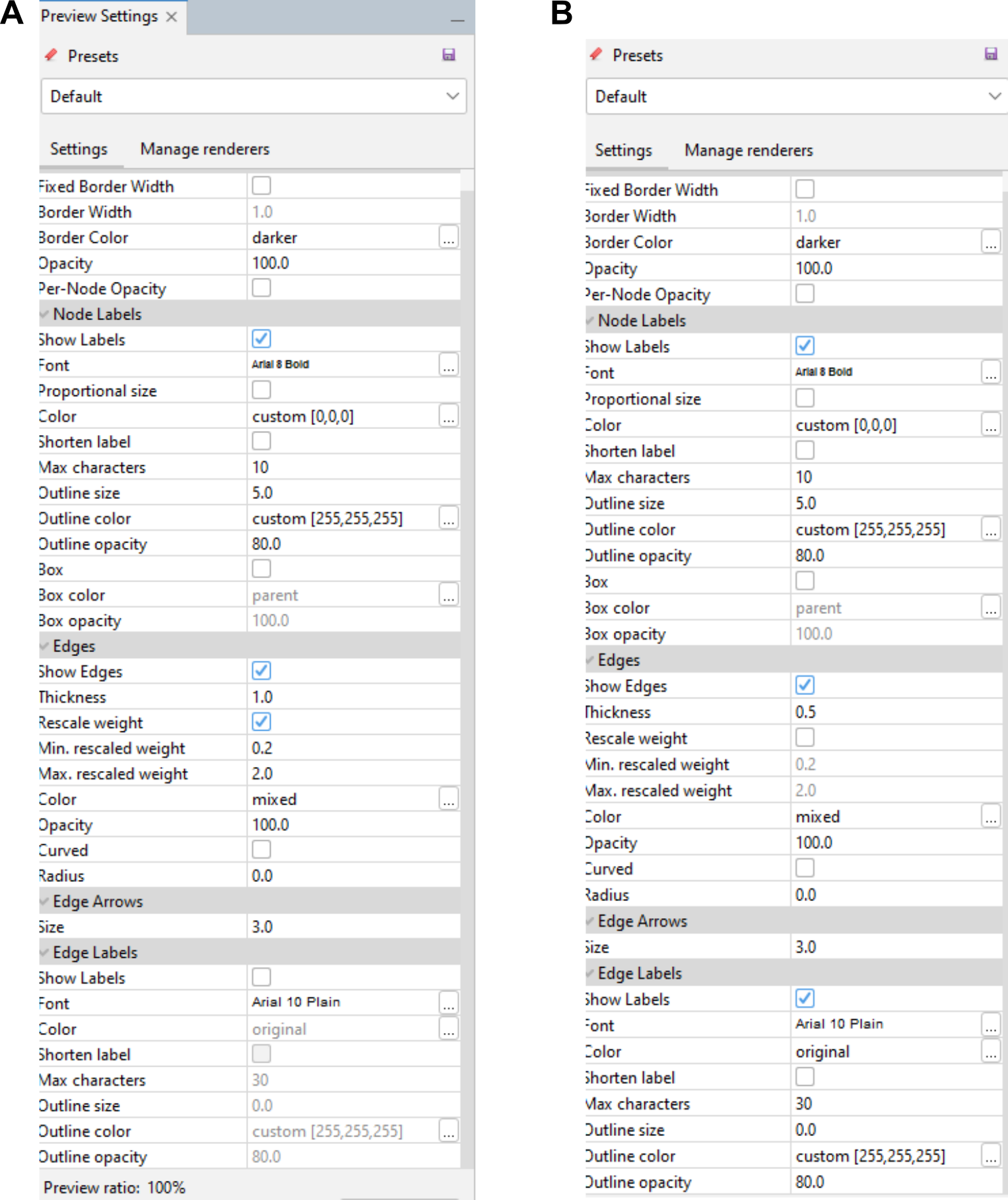


Fig C2: Gephi Preview settings (Bastian M., Heymann S., Jacomy M. (2009). ***Gephi: an open source software for exploring and manipulating networks.*** International AAAI Conference on Weblogs and Social Media.) to visualize the cross-boundary governance network of four neighboring administrative regions on the coast of Pará, North Brazil. A) for the complete network. B) for network parts.

## **D** Council Connection network


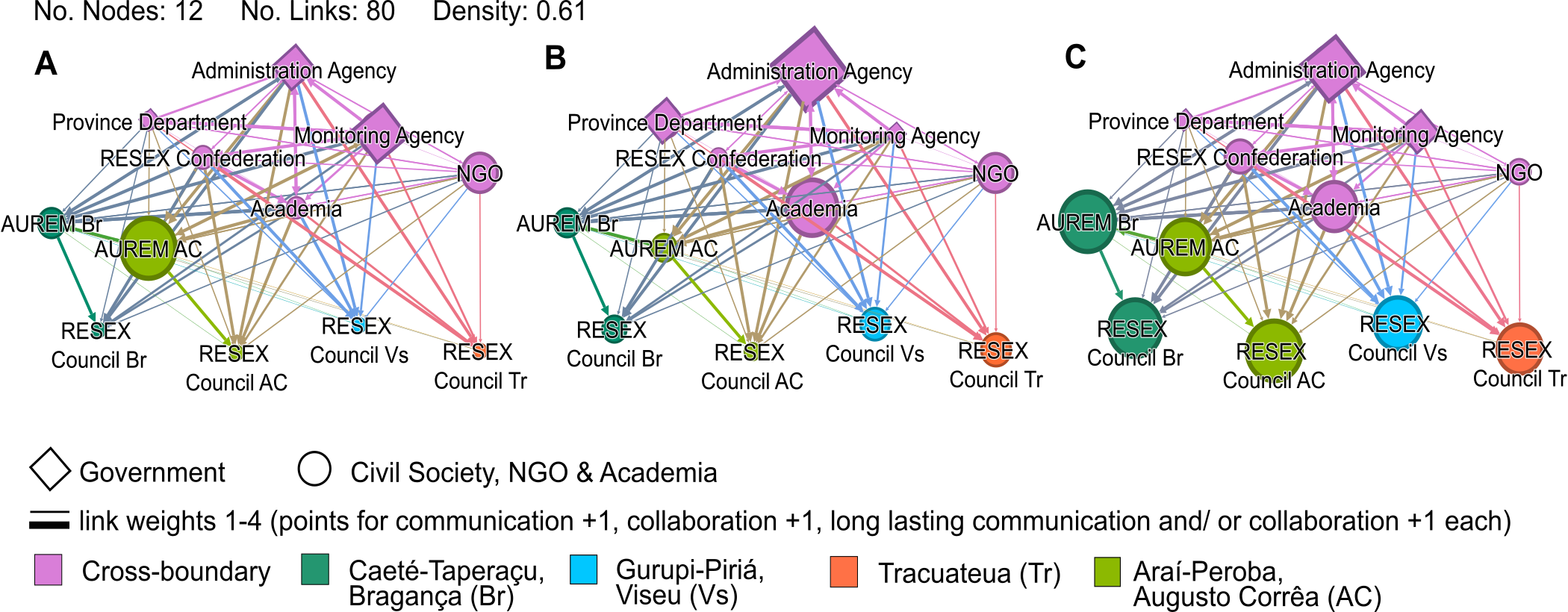


Fig D1. Regional linking of four RESEX Councils in neighboring administrative regions on the coast of Pará, north Brazil. Node sizes represent A Betweenness centrality (Range 0 - 2.483), B Eigenvector centrality (Range 0.505 -Y) and C weighted in-degree (Range X-Y). Link colors are a color mix of sending and receiving node color.

Table D1. Network measures for the regional bridging network of four RESEX Councils in neighboring administrative regions on the coast of Pará, north Brazil. Highest five values per measure highlighted by gray shades and thick box borders.

| **organization** | **betweenness centrality** | **eigenvector- centrality** | **weighted in-degree** |
| --- | --- | --- | --- |
| **regional** | | | |
| Province Department | 0.2 | 0.689 | 7 |
| Monitoring Agency | 1.53 | 0.469 | 13 |
| RESEX Confederation | 0.45 | 0.522 | 14 |
| Administration Agency | 0.817 | 1 | 19 |
| Academia | 0.367 | 0.956 | 20 |
| NGO | 1.333 | 0.754 | 11 |
| **Araí-Peroba, Augusto Corrêa (AC)** | | | |
| RESEX Council AC | 0 | 0.505 | 24 |
| AUREM AC | 2.483 | 0.622 | 22 |
| **Caeté-Taperaçu, Bragança (Br)** | | | |
| RESEX Council Br | 0 | 0.612 | 23 |
| AUREM Br | 0.817 | 0.634 | 24 |
| **Tracuateua (Tr)** | | | |
| RESEX Council Tr | 0 | 0.679 | 20 |
| **Gurupi-Piriá, Viseu (Vs)** | | | |
| RESEX Council Vs | 0 | 0.673 | 21 |

## **E** Initial network interpretation in the focus group discussions

To help understand the displayed network, nodes and links can be given different labels, shapes, colors, and sizes. In this study, the nodes were sized based on betweenness centrality, eigenvector centrality and weighted in-degree, each in separate network maps. Size settings were set to a size minimum of 5 and a size maximum of 20. Node colors indicate the represented organization's region of activity. Link colors arise as a mixture of sending and receiving node color and, therefore, indicate regional or local region connections.


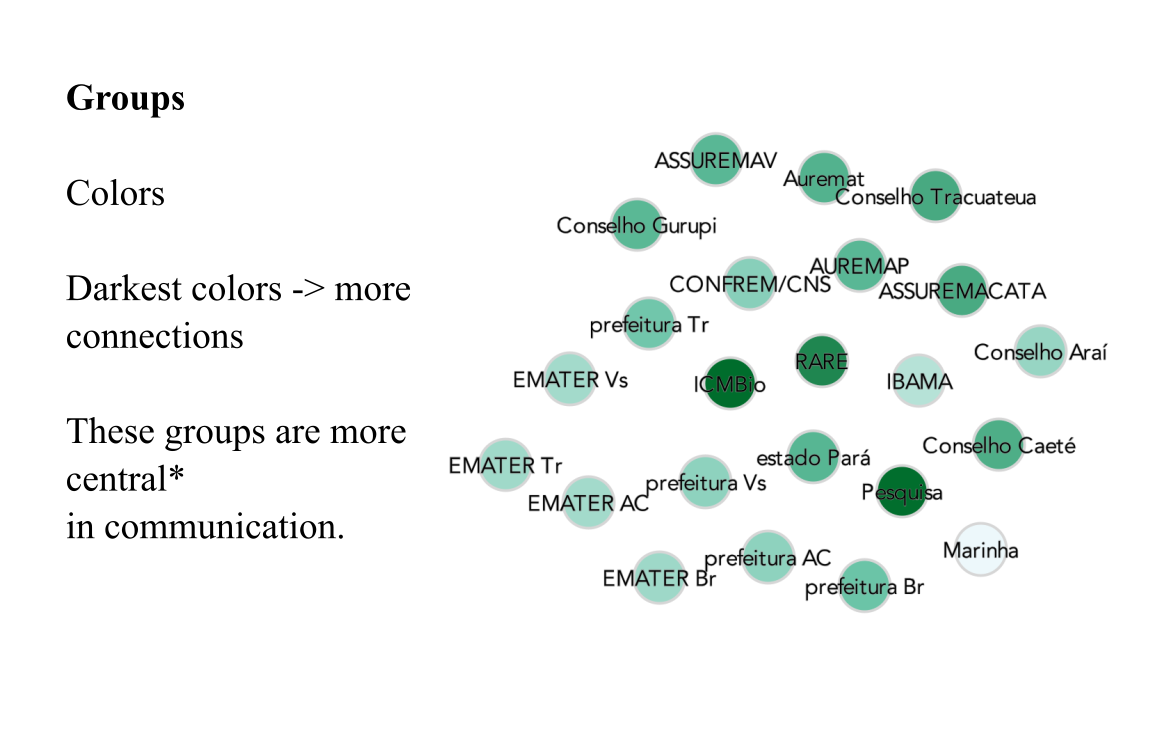


Fig E1. Slide shown to participants of the workshops (in Portuguese) with an explanation about the meaning of the colors and the position of the groups in the network space.

Fig E2. Slide shown to participants of the workshops (in Portuguese) with an explanation about how the councils are not acting as bridges between these groups, which raised the question of how to connect the RESEXs in this setting.





Fig E3. Slide shown to participants of the workshops (in Portuguese) with an explanation about how the councils are not acting as bridges between these groups, which raised the question of what the roles of the AUREMs and other central groups are in connecting the councils and, therefore, the RESEX.

## **F** Extended Interview Excerpts

This section of the SI provides complete quotes from interviews and focus group discussions that support findings presented in the main manuscript. All quotes are presented as originally spoken (translated from Portuguese by the authors) to preserve the authentic voices of stakeholders involved in the governance of extractive reserves (RESEXs) in the Bragança region, Pará, Brazil.

Quotes are organized thematically and referenced in the main manuscript by their SI-F identifier (e.g., SI F1.1, SI F2.3). Interview sources are anonymized to protect participant confidentiality while indicating the stakeholder group represented.

## F1. Resource Constraints and Funding Challenges

### F1.1 Administration Agency on barriers to creating an integrated forum

Source: Administration Agency staff, individual interview

[What could be done to increase the partnership between the different RESEXs, what is missing, is] really this forum [...] This forum that we would like to have [...] needs resources to do, you need to bring people, host people, feed people. So, really, as long as we have the support to do this, in the very project that we presented to [a donor], to try to be included, there is already a line of action along these lines, of managing it, providing the resources for it. But it will depend on whether it is approved. [...] We have a small group of just the presidents [of the AUREMs]. [...]There's a WhatsApp group. From time to time, we get them together here. [...] But to give you an idea, I've already had to pay for gas for one of the presidents out of my pocket because the guy couldn't come; he had to come here. So I said, "Come, and I'll put gas in your motorbike." So, there are very big limitations to doing this intercity thing.

Context: Discussing what is needed for integrated management across the four RESEXs and the practical barriers to implementing a forum of councils.

### F1.2 AUREM president on partnership imbalance with Administration Agency

Source: President of one of the AUREMs, individual interview

What kind of partnership is that? One day, I asked [someone at the Administration Agency]. Well, we go to events, and sometimes we don't have money for a ticket. Damn it. They have a car, they have fuel, they don't do the work in the associations [AUREMs], and they just sit there. Damn it. [...] We need a car, man. We... Everyone is broke here. The [Administration Agency], we're [...] partners. Aren't you going to help?

Context: Interviewee expressing frustration about the lack of material support from the Administration Agency despite being called "partners" in RESEX co-management.

### F1.3 NGO representative on pandemic and political impacts on councils

Source: NGO staff, individual interview

The Administration Agency is now starting to resume its activities. Last year [2021], they spent the whole year distributing food parcels. It was basically the only activity they did, right? Then, this year, they've started talking to the associations [AUREMs] again to resume the agenda of councils, which is something that has been at a complete standstill, I think, since 2019, more or less. I think [it has to do with the pandemic], but with this dismantling that has also happened at the federal level, right? A demobilization, a cut in resources. I think it's a whole situation that has also been added to the pandemic. I think there are several factors. There were also few people working at the NGI, so now we've had new staff come in [from a recent hiring process].

Context: Explaining why RESEX councils became inactive and the multiple factors (pandemic, federal dismantling, resource cuts) that contributed to this situation.

## F2. Digitalization and Communication Barriers

### F2.1 AUREM president on digital skills and infrastructure challenges

Source: President of one of the AUREMs, individual interview

We [the four local RESEXs] need to unite more because we live a long way apart. We have not even learned the first part yet. For example, this pandemic is something that should never happen again. But, on the other hand, there has been a lot that we have learned to do far away, well, and that works. [Over] the internet. But you don't have a good internet connection, you don't have a good device, right?[...] [We don't know] how to handle it. [...] For example, I still haven't wanted to learn... I still haven't bothered to pick up my phone and say, "Look, we're going to hold a meeting with our RESEX here...". Do it over the phone… Then, I'll make a channel and send the link to the people. I don't know how to do that. [...] And there's the issue that it's per minute… [...] Then, for example, if you've signed up, you can use it for 20 minutes, you can use it for half an hour, 1 hour, 2 hours… [...] The one I showed you here, we're doing it. There are 10 minutes left. I'll create another link. But I'll learn. When you come here [next time], I'll [have already learned].

Context: Discussing barriers to cross-RESEX collaboration and the challenges of adopting digital communication tools, including infrastructure limitations, digital literacy gaps, and data costs.

## F3. Administration Agency Role and Perceptions

### F3.1 Administration Agency on their presence and partnerships

Source: Administration Agency staff, individual interview

The Administration Agency's presence has already been felt. We're invited to all the forums. Sometimes there are so many meetings [...]. We even have to excuse ourselves. [...] There is an environmental council here, a fishing council, a council for this, another council for that. [...] We have many partnerships [with researchers]. The partnership with the university has increased a lot. [Since the beginning of the current] administration, it got a lot closer—various branches of research.

Context: Describing the Administration Agency's involvement in regional governance and their strong partnership with academia.

### F3.2 Administration Agency on demand from beneficiaries and their management role

Source: Administration Agency staff, individual interview

Yes, with everything that's happened, [management] is going well. We recognize the increase in demand from the beneficiaries themselves when they need to get wood to renovate their fishing traps. They are already aware that they need to come here for a permit. Someone who builds a little house, you come here to give him a permit, then you do an inspection, then you go there. The municipal entities themselves, nowadays, for any major project… The [municipal] Departments come here to apply for a permit for the project.

Context: Discussing how the Administration Agency is recognized as the authoritative body for issuing permits and how this represents their management role in practice.

### F3.3 AUREM member on who actually manages the RESEX

Source: Member of one of the AUREMs, focus group discussion

Who manages the [RESEX]? It is [the Administration Agency]. It's not the council. The council does not manage it. Decisions are made by the council, but it does not manage [the RESEX].

Context: Clarifying, from the user perspective, that despite the council's theoretical decision-making role, actual management is conducted by the Administration Agency.

### F3.4 Administration Agency on research permit approval processes

Source: Administration Agency staff, individual interview

So, now, the projects that have a significant impact on the communities, then we certainly go through the council. [...] This is a different understanding from a colleague, for example, […] who thinks that all research should go to the council. [...] He thinks that all research should go to the council, and so I think that, if I were to do that [we would be doing] nothing [else in the council meetings]. [We would need] a council meeting every week to approve research. Not us, not the councilors [would be doing anything else than approving research permits].

Context: Defending the current practice of selective council involvement in research permit approvals based on practical constraints and meeting frequency limitations.

### F3.5 Town Department on RESEX-Tr management balance

Source: Town Department Secretary, individual interview

[The RESEX here] is managed by the Administration Agency. For any activity that we [municipal environmental department] are going to do there, we ask for [the Administration Agency's] support because basically the management and organizational part of the RESEX is done by the association [AUREM] and by the Administration Agency.

Context: Describing RESEX-Tr as an example where management is more balanced between the Administration Agency and the AUREM, representing a better working relationship.

## F4. AUREM Functioning and Representation

### F4.1 Town Department on AUREM management problems

Source: Town Department Secretary, individual interview

The association [AUREM] itself is a problem in society. But why is it a problem if the association is supposed to be a solution? It is a problem when creating an association. The association has to have a president or someone on the board who at least knows how to manage it, right? If you don't know how to manage it, the association is created after a year, and then it starts having problems because there's a whole process to follow. And people simply don't know how to create because they think that a group of people together can do more things - they can raise funds, raise projects, raise this? They don't worry about the future, and then when they get there, the association is in default, it has people with bad names and lots of other things. So I think that the administration and the association's management really need to be linked to these issues of... of the president himself, for example.

Context: Criticizing the lack of management capacity in AUREMs and the problems that arise from inadequate organizational skills.

### F4.2 Agricultural Assistance on AUREM centralization

Source: Agricultural Assistance staff, individual interview

Today, we see [the AUREMs] centered on just one person. We don't see many more people involved, which, in my view, there are. There are many areas here. [One of the RESEXs] is very representative, and the RESEX is in the hands of just one person. That's what we hear. [...] It's very bad. The RESEX has to be something participatory; it has to have a front with the public entities with [Agricultural Assistance], state, municipal, the associations, the communities, the local leaders, [...] etc.

Context: Expressing concern that AUREMs are overly centralized around individual presidents rather than being truly participatory organizations.

## F5. Tensions Between Administration Agency and AUREMs

### F5.1 AUREM member on government attempting to control councils

Source: Member of one of the AUREMs, focus group discussion

[...] Today we see a lot of difficulty [...] In a way, the Administration Agency imposes itself in order to say, for example, that the town hall... The town hall has a seat. There should be a seat that they can have, which has always been naturally the [Environmental] Town Department. Today, the Administration Agency is trying to impose that the Town Department has one seat and the town hall has another. And this is an affront to us. And possibly they are creating the Fishing Department. Maybe they want another seat. [...] It seems that they are trying to pull in bodies from within the municipality. Government bodies. [...] This is a... It is a very big risk. And we realize that this clash is always endorsed, wanting to be endorsed by the Administration Agency. And this is a very big risk for us, with actions that border on authoritarianism, bad intentions, and a lack of institutional character.

Context: Expressing fear during the focus group that the Administration Agency is attempting to populate councils with government bodies to dilute local user representation and decision-making power.

### F5.2 AUREM president on research permit control

Source: President of one of the AUREMs, focus group discussion

(1) We're not against research. We welcome all research as long as the association [AUREM], the board, and the people in the community agree.

(2) We are partners of the [local university], but [the relationship] is kind of scratched.

Context: (1) Asserting that AUREMs want approval authority over research permits; (2) Expressing dissatisfaction with the university relationship, related to lack of research "return" (giving back/reaching out) to communities.

## F6. NGO Partnerships and Collaboration

### F6.1 Town Department on NGO's role bringing groups together

Source: Town Department Secretary, individual interview

[When I took office] I did not know anyone from the other reserves, but then [the NGO] came and built the COASTAL500. And they formed these groups and now it is better. Today we have better information about the other reserves. Even in Soure, which is a long way away, we already have some information [about it]. So I think we should keep going. I think we're on the right track. [The NGO] always puts us together. They bring us together. [The NGO's] role is very much to unite us with other institutions.

Context: Praising the NGO's role in facilitating cross-RESEX connections and information sharing, particularly through the COASTAL500 project.

### F6.2 AUREM president on evolving partnership with NGO

Source: President of one of the AUREMs, individual interview

[The NGO] puts in its thoughts, and we put in ours. Because [...] I discuss the issue of partnership with them. What is a partnership? A partnership is when we have something in common, and I contribute x, and you contribute x. [...] When you contribute and want to be in charge, it is different. [...] When I joined, it was when [the NGO] arrived with a program. And then they said, "No, because we want it that way...". [I said], "No, wait a minute. I do not want your program. If you want to help me, we have a proposal, which I was getting into at the time. [...] But it was a good thing because we started getting together again. The question came up again... And today, we're meeting in committees, we are reorganizing our base. And I thanked [the NGO]. I said at the meeting that I appreciated the help. And having this partnership means you also respect the other partner.

Context: Describing initial tensions with the NGO over agenda-setting and how open dialogue led to a more balanced partnership based on mutual respect, ultimately producing positive outcomes for the AUREM.

## F7. Academic Relationships and Research Return

### F7.1 AUREM member on importance of research and need for return

Source: Member of one of the AUREMs, focus group discussion

There are many professors, researchers who are playing their part. [...] It's a benefit because of the fishermen and everything else. I have to give it back to them because if you don't have research, you cannot survive. [...] And research is very important to us. We need to keep this in mind, that our university… [it needs] to put what we want to learn back into our community, into the society.

Context: Acknowledging the value of academic research while emphasizing the need for researchers to return results to the community in meaningful ways - both sharing findings and applying research to improve local conditions.

### Notes on Translation and Anonymization

- All quotes were originally spoken in Portuguese and translated by the authors
- Stakeholder positions are indicated by general category (e.g., "Administration Agency staff," "AUREM president") rather than names to protect confidentiality
- Bracketed text [...] indicates omitted portions for brevity or clarity
- Bracketed text [word] indicates translator clarification or context
- Some identifying details (e.g., specific project names, individual names) have been replaced with generic descriptors [in brackets] to preserve anonymity

## **G** Additional Context on Governance Challenges

### G1 Detailed permit statistics

Referenced in: Section 3.4.3 of the manuscript

The Sisbio platform shows that 66, 174, 80, and 67 permits have been granted for the RESEXs AC, Br, Tr, and Vs, respectively, from 2007 to 2024. This corresponds to an average of approximately 4-10 projects per year per RESEX.

According to available meeting minutes for RESEX-Br from 2006 to 2020, the annual frequency of ordinary council meetings varies from 0 (zero) to 5 (five). Therefore, with an average of 10 permits and, in the best-case scenario, 5 council meetings per year, at least 2 permit requests would need to be discussed in each RESEX council meeting.

Context: This calculation supports the Administration Agency's argument that bringing all research permits to council meetings would be operationally challenging given the technical nature of proposals and limited meeting frequency.

## **H** Focus group composition details

Referenced in: Section 2.4 of main manuscript

Focus Group 1 (RESEX-Br): 9 attendees - AUREM president, representatives from village hubs, and other invited guests

Focus Group 2 (RESEX-AC): 5 attendees - AUREM leadership and village representatives

Focus Group 3 (RESEX-Vs): 8 attendees - AUREM council members and community leaders

**I** Detailed Network Centrality Measures (Full Data for Table 1)

This section provides the complete quantitative results from the Social Network Analysis (SNA) utilized in the main manuscript, following the condensation of results requested by reviewers. Table I1 presents the values for the three core network centrality measures—Betweenness Centrality, Eigenvector Centrality, and Weighted In-degree—for all 23 organizations included in the study. The measures are displayed across the three network scenarios defined in the Methods (Section 2.3): the Whole Network (all actors included), the network without RESEX Councils, and the network without RESEX Councils and AUREMs. These data support the findings and interpretations presented in the Main Text Section 3.1 regarding the centrality, bridging, and influential capacity of key governance actors.

Table I1. Network measures of organizations involved in the governance of four RESEXs, on the coast of Pará, north Brazil.

| **organization** | **whole network** | | | **without RESEX Councils** | | | **without RESEX councils and AUREMS** | | |
| --- | --- | --- | --- | --- | --- | --- | --- | --- | --- |
|  | **betweenness centrality** | **eigenvector- centrality** | **weighted in-degree** | **betweenness centrality** | **eigenvector- centrality** | **weighted in-degree** | **betweenness centrality** | **eigenvector- centrality** | **weighted in-degree** |
| **regional** | | | |  | | |  | | |
| Province Department | 21.915 | 0.689 | 40 | 14.3274 | 0.689 | 40 | 8.3 | 0.689 | 38 |
| Monitoring Agency | 12.045 | 0.469 | 20 | 10.151 | 0.469 | 20 | 6.246 | 0.469 | 13 |
| RESEX Confederation | 6.1397 | 0.522 | 17 | 5.302 | 0.522 | 17 | 1.5 | 0.522 | 11 |
| Administration Agency | 34.719 | 1 | 54 | 26.655 | 1 | 54 | 19.2095 | 1 | 44 |
| Academia | 32.157 | 0.956 | 49 | 25.260 | 0.956 | 49 | 17.585 | 0.956 | 40 |
| Navy | 0.536 | 0.370 | 18 | 0.536 | 0.370 | 18 | 0.6 | 0.370 | 14 |
| NGO | 8.992 | 0.754 | 23 | 5.612 | 0.754 | 23 | 1.7 | 0.754 | 15 |
| **Araí-Peroba, Augusto Corrêa (AC)** | | | |  | | |  | | |
| Town Department AC | 2.248 | 0.620 | 22 | 2.248 | 0.620 | 22 | 2.504 | 0.620 | 22 |
| RESEX Council AC | 0 | 0.505 | 24 | - | - | - | - | - |  |
| AUREM AC | 5.406 | 0.622 | 31 | 3.719 | 0.621 | 31 | - | - | - |
| Agricultural Assistance AC | 3.343 | 0.516 | 30 | 3.343 | 0.516 | 30 | 1.940 | 0.516 | 28 |
| **Caeté-Taperaçu, Bragança (Br)** | | | |  | | |  | | |
| Town Department Br | 6.495 | 0.673 | 25 | 5.928 | 0.673 | 25 | 5.004 | 0.673 | 24 |
| RESEX Council Br | 0 | 0.612 | 29 | - | - | - | - | - | - |
| AUREM Br | 5.259 | 0.634 | 31 | 3.912 | 0.634 | 31 | - | - | - |
| Agricultural Assistance Br | 3.177 | 0.522 | 31 | 2.393 | 0.522 | 31 | 2.003 | 0.522 | 28 |
| **Tracuateua (Tr)** | | | |  | | |  | | |
| Town Department Tr | 3.735 | 0.682 | 24 | 3.225 | 0.682 | 24 | 3.091 | 0.682 | 22 |
| RESEX Council Tr | 0 | 0.679 | 30 | - | - | - | - | - | - |
| AUREM Tr | 9.359 | 0.734 | 35 | 9.073 | 0.734 | 35 | - | - | - |
| Agricultural Assistance Tr | 3.408 | 0.531 | 30 | 2.708 | 0.531 | 30 | 1.661 | 0.531 | 28 |
| **Gurupi-Piriá, Viseu (Vs)** | | | |  | | |  | | |
| Town Department Vs | 7.677 | 0.677 | 25 | 7.110 | 0.677 | 25 | 6.804 | 0.677 | 24 |
| RESEX Council Vs | 0 | 0.673 | 33 | - | - | - | - | - | - |
| AUREM Vs | 3.456 | 0.678 | 36 | 3.344 | 0.678 | 36 | - | - | - |
| Agricultural Assistance Vs | 3.934 | 0.526 | 29 | 3.150 | 0.526 | 29 | 1.853 | 0.526 | 28 |

1. de Juan S, Ospina-Alvarez A, Castro AJ, Fernández E, Méndez-Martínez G, Molina J, et al. Understanding socioecological interaction networks in Marine Protected Areas to inform management. Ocean & Coastal Management. 2023;245: 106854. doi:[10.1016/j.ocecoaman.2023.106854](https://doi.org/10.1016/j.ocecoaman.2023.106854) [↑](#footnote-ref-2)
2. https://issuu.com/rarebrasil/docs/rarebrasil_relatorioexecutivo2017-2019 [↑](#footnote-ref-3)
