# Supplementary material for: Bridges or Barriers? Cross-boundary communication and governance mismatches in co-managed protected areas
Source: PLoS One. 2026 Apr 6;21(4):e0342438. doi: 10.1371/journal.pone.0342438 (PMC13052881; doi:10.1371/journal.pone.0342438)
Supplement: Attachment — Submitted filename: Reply to Reviewer 2.docx [file pone.0342438.s003.docx]

Dear Reviewer 2,

Thank you so much for your thorough and thoughtful review. We very much appreciate your feedback and the time you have dedicated to evaluating our manuscript.

Below we address the points raised during the review process and hope we have approached them in a proper manner.

Yours sincerely

The authors

***Specific replies to comments:***

*Thank you for the opportunity to review your manuscript, which presents an insightful and original investigation into cross-boundary communication and governance mismatches in Brazilian coastal-marine Extractive Reserves (RESEXs). Your use of social network analysis (SNA) in combination with qualitative data offers a rich, multidimensional view of how institutional actors function within and across protected area governance landscapes.
The research is timely, methodologically thoughtful, and conceptually rigorous. It contributes meaningfully to scholarship on co-management, marine spatial planning, and the socio-institutional dimensions of environmental governance—particularly within the under-researched context of community-managed marine protected areas in the Global South.
This is a high-quality manuscript that presents novel, methodologically sound, and theoretically important findings.*

**Reply**: Thank you for this generous and encouraging assessment of our work. We are honored by your recognition of our interdisciplinary approach and are so grateful to have the opportunity to contribute meaningfully to this important field of research. We deeply appreciate your support and esteem of our work.

*To enhance its impact and ensure full compliance with journal requirements, I recommend minor revisions, particularly in relation to:*

*• Ethical declarations and data availability transparency.*

**Reply:** We confirm that all ethical declarations are included in the designated Ethics Statement section of the manuscript. Our research received ethics approval from the University of Oldenburg (Drs.Nr.EK/2022/053) and research permission from Sisbio (82744-1). All participants provided written and/or oral informed consent. Regarding data availability, we have deposited the anonymized network data and supporting materials on Zenodo (DOI: 10.5281/zenodo.17241755). Raw interview transcripts have not been shared to protect participant confidentiality, as per our ethics protocol. We have ensured that the data availability statement in the manuscript reflects this information.

*• Methodological detail, particularly regarding interview sampling and SNA metrics.*

**Reply**: We appreciate this feedback and have enhanced the methodological detail in the following ways:

Interview sampling: Previously, we had clarified in the Methods section (2.2) that we used purposive sampling to identify key stakeholder groups relevant to cross-boundary RESEX governance, with the actor list compiled from publicly available council composition decrees and a regional management database. Our sampling focused on groups that were current or past council members, had strong interest in participating in council governance, or wished to have their interests considered by the management councils.

We added the mention that the four RESEX councils themselves were included as potential targets of interaction in the SNA survey but were not interviewed as separate entities, since council members already represented their respective organizations in our sample (each council member belongs to one of the organizations surveyed). We have specified the recruitment periods (November-December 2022 and April-May 2024) and detailed our inclusion/exclusion criteria, including the removal of groups that no longer existed, had lost interest in RESEX governance, or were specific to only one RESEX (as our focus was on regional cross-boundary management). See section “2.2 Selected social groups for social network analysis” of the new document version with markings

SNA metrics: We have expanded our explanation of the specific SNA metrics employed (section 2.3, "Analyzed network parameters", line 548 of the new document version with markings). These include: (a) betweenness centrality (directed, normalized) to assess brokerage capacity and identify nodes facilitating communication between network parts; (b) eigenvector centrality (directed, 100 iterations, 0-1 scale) to measure node importance based on connections to other influential nodes; and (c) weighted in-degree to capture both the number and quality of incoming connections, with weights (0-4) assigned based on interaction type (communication and/or collaboration) and duration (long-lasting vs. recent). We have clarified why we used in-degree rather than out-degree (the RESEX councils could not be interviewed as separate entities) and provided the rationale for our weighting scheme. The specific Gephi layout parameters (ForceAtlas2 and LabelAdjust settings) are detailed in Supporting Information C.

*• Minor enhancements to figures and formatting for accessibility.*

**Reply**: Images have been improved, with a minimum resolution of 300dpi, as well as other improvements. Thank you for pointing this out.

*I commend your interdisciplinary and participatory approach to understanding marine governance systems, and I am confident that, with these revisions, the manuscript will make a valuable contribution to both academic literature and practical conservation policy.*

**Reply**: Thank you so much for your appreciation of our approach and for your confidence in our work's potential impact.

*Thank you again for your important work and for submitting your manuscript to PLOS ONE. I hope these comments are helpful as you prepare your revised submission.*

**Reply**: We very much receive your feedback with gratitude and are happy to get the chance to contribute to academic literature, as well as practical conservation policy. We look forward to submitting our revised manuscript to PLOS ONE and sincerely appreciate the constructive guidance you have provided.
